# Association of Mitochondrial Genetic Variation with Carotid Atherosclerosis

**DOI:** 10.1371/journal.pone.0068070

**Published:** 2013-07-09

**Authors:** Igor A. Sobenin, Margarita A. Sazonova, Anton Y. Postnov, Jukka T. Salonen, Yuri V. Bobryshev, Alexander N. Orekhov

**Affiliations:** 1 Russian Cardiology Research and Production Complex, Moscow, Russia; 2 Institute of General Pathology and Pathophysiology, Moscow, Russia; 3 MAS-Metabolic Analytical Services Oy, Helsinki, Finland; 4 University of Helsinki, Hjelt Institute, Helsinki, Finland; 5 Institute for Atherosclerosis Research, Skolkovo Innovation Center, Moscow, Russia; 6 Faculty of Medicine, University of New South Wales and St Vincent’s Hospital Sydney, Kensington, New South Wales, Australia; Innsbruck Medical University, Austria

## Abstract

In human pathology, several diseases are associated with somatic mutations in the mitochondrial genome (mtDNA). Even though mitochondrial dysfunction leads to increased oxidative stress, the role of mitochondrial mutations in atherosclerosis has not received much attention so far. In this study we analyzed the association of mitochondrial genetic variation with the severity of carotid atherosclerosis, as assessed by carotid intima-media thickness (cIMT) and the presence of coronary heart disease (CHD) in 190 subjects from Moscow, Russia, a population with high CHD occurrence. cIMT was measured by high-resolution B-mode ultrasonography and mtDNA heteroplasmies by a pyrosequencing-based method. We found that heteroplasmies for several mutations in the mtDNA in leukocytes, including C3256T, T3336C, G12315A, G13513A, G14459A, G14846A, and G15059A mutations, were significantly (p<0.001) associated with both the severity of carotid atherosclerosis and the presence of CHD. These findings indicate that somatic mitochondrial mutations have a role in the development of atherosclerosis.

## Introduction

In human pathology, several diseases have been associated with somatic mutations in the mitochondrial genome. These mitochondrial mutations may arise during ontogenesis and are associated with pathologies such as coronary vessel stenosis, some forms of diabetes, myocardial infarction, cardiomyopathy and other pathologies [1]–[17].

Atherosclerosis, the most common pathology in modern society, is a multifactorial disease, in the development and progression of which an interaction of phenotypic, environmental, socioeconomic and genetic factors plays a significant role. Numerous polymorphisms of the nuclear genome, which are believed to be genetic risk factors for atherosclerotic diseases, can help to explain for only a few percentages of the variability of clinical manifestations of atherosclerosis, such as coronary heart disease (CHD). At the same time, mutations of the mitochondrial genome have remained out of focus for a long time. However, they may play a pathogenic role in the formation of atherosclerotic lesions of human arteries causing various defects in the protein chains of some energy-generating enzymes and transfer RNA (tRNA), synthesized directly in the mitochondria. This leads to a decrease in the concentration of these enzymes and their tRNA or total dysfunction, which contributes to the development of oxidative stress, deterioration of ATP production and accelerated development of atherosclerosis.

In 2009, we developed a mutant allele quantitative assay to study the differences in tissue-specific mitochondrial heteroplasmies [18]. Using this methodology we showed that there are significant differences between unaffected intima and atherosclerotic lipofibrous plaque in the level of heteroplasmy for several point substitutions [18]. Further analysis revealed that several mutations could be found in intimal cells that populate atherosclerotic lesions [19].

**Figure 1:**
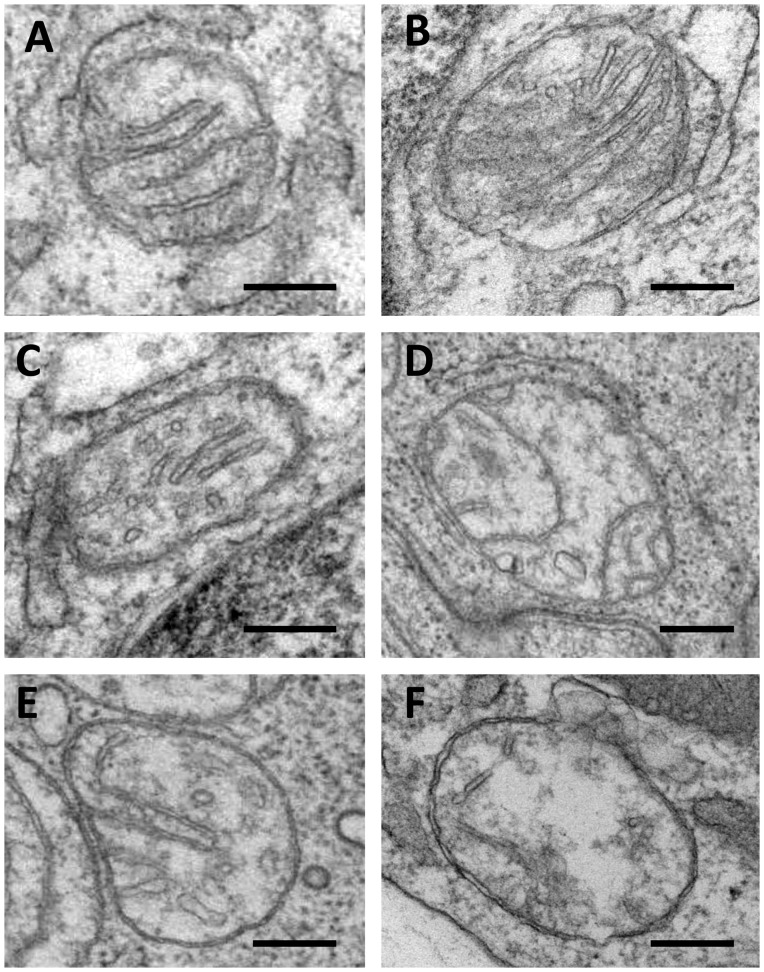
Different ultrastructural appearances of mitochondria in leukocytes obtained from healthy volunteers and patients with carotid atherosclerosis (A–F). (**A**): A mitochondrion with well-defined cristae and well-preserved surrounding membranes typically seen in leukocytes of healthy volunteers. (**B–F**): Mitochondria with reduced numbers of cristae and the oedema of the mitochondrial matrix observed in patients with carotid atherosclerosis (**A–F**): Electron microscopy. Scales = 150 nm.

**Figure 2:**
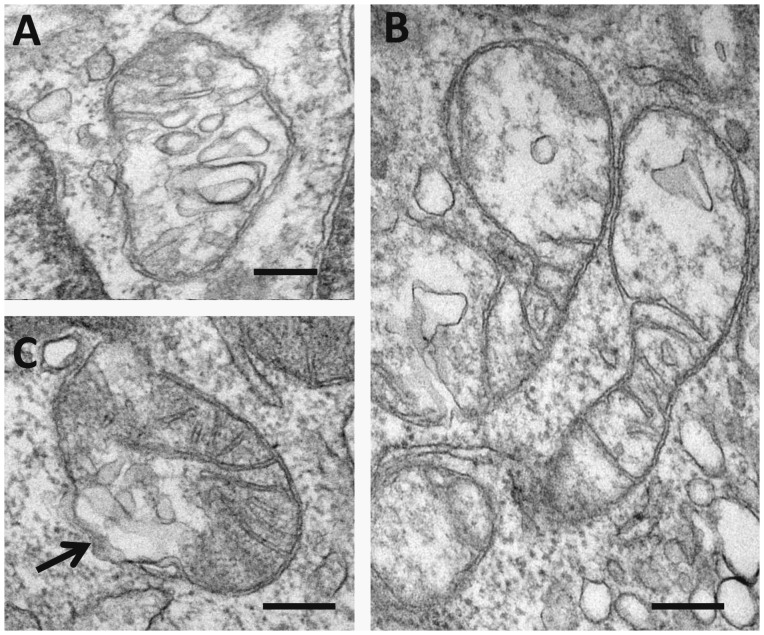
Ultrastructural appearances of mitochondria in leukocytes obtained from patients with carotid atherosclerosis (A–C). In (**A, B**), note a profound focal oedema of the mitochondrial matrix and destructive alterations of cristae. In (**C**), a zone of destruction of the outer mitochondrial membrane in shown by arrow (**A–C**): Electron microscopy. Scales = 150 nm.

In a pilot study of ultrastructural characteristics of leukocytes (Text File S1) we noted that in subjects without carotid atherosclerosis, mitochondria typically contained well-defined cristae, which were regularly located in the mitochondria matrix, but the distribution and location of cristae in mitochondria in leukocytes obtained from the blood of subjects with carotid atherosclerosis were different (Fig. 1A–F). In some leukocytes obtained from the blood of patients with carotid atherosclerosis destructive alterations of mitochondria that were not present in leukocytes of healthy subjects were observed (Fig. 2A–C). This observation motivated us to investigate possible presence of mutations in leukocytes in carotid atherosclerosis; The analysis of mutation C3256T has revealed that there is a relation between C3256T heteroplasmy level and predisposition to atherosclerosis [20].

These findings motivated us to undertake the present study, which was aimed to investigate if there might be association of the levels of heteroplasmy of other mutations in leukocytes with the extent of carotid atherosclerosis as well as the presence of CHD. In the present study a spectrum of well-known mutations, such as A1555G, C3256T, T3336C, C5178A, G12315A, G13513A, G14459A, G14846A, and G15059A, was analyzed.

## Results

The phenomenon of heteroplasmy was observed for all examined mutations of mitochondrial genome in the vast majority of DNA samples, although the profiles of distribution differed between mutations (Table 1). (In the present report, in order that the levels of heteroplasmy among different mutations were visible, we also included analysis of mutation C3256T, the data about which was reported earlier [20]). The presence of mutation A1555G was not detected only in one sample of 190, T3336C – in 3 samples, C5178A – in 6 samples, and G13513A – in 9 samples. All other mutations were detected in all samples. Additionally, 100% of mutant allele was detected in DNA from blood leukocytes only in one sample for T3336C, and in two samples for the G15059A mutations.

**Table 1:** Characteristics of distributions of heteroplasmy levels.

Mutation of mtDNA	Heteroplasmy level, %					The proportion of cases with identified mutation, %
	range	25^th^ percentile	median	75^th^ percentile	mean (SD)	
A1555G	0–83	10	13	18	16,5 (10.9)	99,5
C3256T	5–74	13	18	36	23.3 (14.7)	100
T3336C	0–100	5	7	10	8.3 (8.3)	98.4
C5178A	0–83	11	15	19	15.6 (10.6)	96.9
G12315A	2–88	18	28	51	32.3 (19.4)	100
G13513A	0–85	10	20	35	23.7 (18.7)	95.3
G14459A	3–82	12	18	33	27.2 (21.1)	100
G14846A	3–96	8	10	15	15.8 (17.4)	100
G15059A	4–100	26	39	47	37.6 (16.8)	100

We found significant correlations between carotid intima-media thickness (cIMT) and the levels of heteroplasmy for C3256T (r = 0.362, p<0.001), T3336C (r = 0.152, p = 0.036), G12315A (r = 0.306, p<0.001), G13513A (r = −0.357, p<0.001), and G15059A (r = 0.316, p<0.001) mutations of the mitochondrial genome (mtDNA). The levels of heteroplasmy for A1555G, C5178A, G14459A, and G14846A had no associations with cIMT. The data on the mean levels of heteroplasmy in the 1^st^ and 4^th^ quartiles of cIMT adjusted for age and gender are presented in Table 2.

**Table 2:** The levels of heteroplasmy of mitochondrial genome of human blood leukocytes in study participants belonging to 1^st^ and 4^th^ quartiles of cIMT distributions, in participants with or without atherosclerotic plaques in carotid arteries, and in those with or without coronary heart disease.

Mutation of mtDNA	Heteroplasmy level, %		P (Mann-Whitney)	Heteroplasmy level, %		P (Mann-Whitney)	Heteroplasmy level, %		P (Mann-Whitney)
	1^st^ quartile cIMT, n = 51	4^th^ quartile cIMT, n = 44		Plaque absent, n = 84	Plaque present, n = 106		CHD absent, n = 145	CHD present, n = 45	
A1555G	19.2 (14.0)	16.9 (11.1)	NS	18.3 (11.3)	15.1 (10.4)	NS	16.8 (11.3)	15.5 (9.5)	NS
C3256T	16.6 (11.3)	28.3 (14.8)	<0.001	16.1 (5.3)	28.8 (17.1)	<0.001	21.6 (13.4)	28.8 (17.3)	0.031
T3336C	7.8 (13.8)	9.4 (4.0)	<0.001	7.3 (10.8)	9.1 (5.6)	<0.001	7.9 (8.7)	9.6 (6.7)	0.019
C5178A	12.2 (7.0)	15.5 (7.5)	NS (0.09)	16.5 (12.5)	14.9 (8.9)	NS	15.5 (10.4)	16.2 (11.3)	NS
G12315A	24.7 (17.1)	39.4 (18.9)	<0.001	24.3 (12.0)	39.1 (21.5)	<0.001	30.9 (18.9)	38.5 (20.2)	0.030
G13513A	30.8 (20.9)	18.3 (20.6)	<0.001	32.1 (17.4)	17.3 (17.2)	<0.001	25.3 (18.4)	18.8 (19.2)	0.006
G14459A	29.8 (24.5)	24.3 (18.6)	NS	28.5 (21.7)	26.2 (20.7)	NS	29.0 (21.9)	21.4 (17.3)	0.018
G14846A	17.8 (21.0)	16.0 (17.2)	NS	15.4 (15.3)	16.1 (18.9)	NS	16.1 (17.5)	14.7 (17.2)	NS
G15059A	33.9 (17.9)	45.0 (19.3)	0.007	33.4 (16.0)	40.8 (16.7)	<0.001	37.6 (16.8)	37.8 (16.8)	NS

Data are presents as mean (SD).

Additionally, the levels of heteroplasmy for mutations C3256T, T3336C, G12315A, G13513A, G14459A, G14846A, and G15059A correlated significantly with the size of atherosclerotic plaques in any visualized segment of carotid arteries, as it was evaluated by 4-point scale (Spearman’s Rho, 0.317, p<0.001, 0.328, p<0.001, 0.356, p<0.001, −0.492, p<0.001, −0.150, p = 0.038, −0.153, p = 0.034, and 0.210, p = 0.003, respectively). The levels of heteroplasmy for A1555G and C5178A did not correlate with the size of atherosclerotic plaques. The data on the mean levels of heteroplasmy in study participants without atherosclerotic plaques and those participants who had an atherosclerotic plaque in any visualized segment of carotid arteries are shown in Table 2.

Of the conventional coronary risk factors, age had the strongest association with the studied mtDNA heteroplasmies. Age correlated with the level of heteroplasmy for C3256T (r = 0.279, p<0.001), C5178A (r = 0.199, p = 0.006), G12315A (r = 0.255, p<0.001), G13513A (r = −0.363, p<0.001), G14459A (r = −0.192, p = 0.008), and G15059A (r = 0.328, p<0.001) mutations of mitochondrial genome of circulating leukocytes. For four of the nine mutations (C5178A, G1231A, G13513A and G14459A) there was a difference between men and women (data not shown). A significant correlation was also found between systolic blood pressure and the G15059A heteroplasmy level (r = 0.218, p = 0.002), and between triglycerides and T3336C (r = 0.291, p<0.001) and G12315A (r = 0.153, p = 0.034) heteroplasmies. None of the studied heteroplasmies correlated with either diastolic blood pressure, serum total cholesterol or LDL cholesterol.

We have performed a regression analysis, in which the positive or negative correlation was factored for deriving the association between mtDNA mutations and atherosclerosis. The model employing multiple regression caused serious problems, since the number of variables that should be included for the construction of a general model was too large for a given sample size. In this case, a serious bias resulted in the loss of statistical significance for all variables, both conventional risk factors and genetic markers. Therefore, each mutation was tested separately for mediation/moderation. For this purpose, paired regression model was estimated (e.g., cIMT vs. mutation, or the presence/absence of atherosclerotic plaque vs. mutation), and then the multiple regression model was built, which also included conventional risk factors, to avoid false correlations. For this analysis, cIMT and the levels of heteroplasmy were taken as quantitative values (not quartile or ranked values). Since cIMT was distributed normally, the assumptions for linear regression analysis have been met. Two models were compared to assess whether the inclusion of mutations adds a significant contribution to the increase of explanatory power for cIMT variability. The first model included only conventional risk factors (age, gender, diabetes, hypertension, triglycerides, LDL cholesterol, HDL cholesterol), and the second model also included the levels of heteroplasmy for mtDNA mutations. The residues in the second model were significantly lower than those in in the model employing only conventional risk factors (Fisher’s statistics 5.09, p<0.001). The model, which included both conventional risk factors and mutations provided significantly better explanatory level than the first one (adjusted R^2^, 33.5% vs. 24.5%). The similar approach was used to assess whether the inclusion of mutations adds a significant contribution to the increase of explanatory power for diagnostics of the presence of atherosclerotic plaque in any visualized segment of carotids. In this case, binary logistic regression analysis was performed, and a direct comparison of logit models was made using chi-square statistic. Mutations provided statistically significant information for diagnostics (p<0.001). So, in-depth statistical analysis have supported our assumption on significant association for atherosclerosis and CHD with heteroplasmy level of both proatherogenic and atheroprotective mtDNA mutations.

It is notable that the levels of heteroplasmy for those mutations, which were correlated significantly with cIMT and/or atherosclerotic plaque size, also correlated between each other (Table 3). As a rule, the levels of heteroplasmy positively correlated with carotid atherosclerosis, were also directly associated with each other, but correlated negatively with heteroplasmy levels of mutations, which had a negative association with atherosclerosis. These correlations suggested the presence of linkage disequilibrium; therefore, we used one more measure of “integral mutation burden of mitochondrial genome”, which was calculated as a simple arithmetic sum of quartile numbers (ranks) of heteroplasmy levels, assigned from the analysis of heteroplasmy distribution within the sample (interquartile borders are shown in Table 1). If heteroplasmy level was positively correlated with carotid atherosclerosis, its quartile number was taken with positive sign, for negative correlations – with negative sign. The measure of integral mutation burden varied from −7 to 11, with a median value of 2. The integral mutation burden correlated significantly with both cIMT (Spearman’s Rho, 0.376, p<0.001) and the size of atherosclerotic plaque (Spearman’s Rho, 0.487, p<0.001).

**Table 3:** Correlations between the levels of heteroplasmy of mitochondrial genome from human blood leukocytes.

Mutation	Pearson’s correlation coefficient					
	T3336C	G12315A	G13513A	G14459A	G14846A	G15059A
C3256T	0.303, p<0.001	0.792, p<0.001	−0.626, p<0.001	−0.435, p<0.001	−0.153, p = 0.034	0.214, p = 0.003
T3336C	–	0.412, p<0.001	NS	NS	NS	NS
G12315A		–	−0.447, p<0.001	−0.363, p<0.001	NS	0.212, p = 0.003
G13513A			–	0.362, p<0.001	NS	−0.238, p = 0.001
G14459A				–	0.164, p = 0.023	NS
G14846A					–	0.196, p = 0.006

The measure of “integral atherosclerotic burden” was also used for analysis; it was calculated as a simple arithmetic sum of quartile numbers of cIMT (adjusted for age and gender) and the score of the size of atherosclerotic plaques in any visualized segment of carotid arteries. Two measures, integral mutation burden and integral atherosclerotic burden, also were correlated significantly (Spearman’s Rho, 0.405, p<0.001). One-way analysis of variation confirmed the strong relationship between these two measures (F = 4.595, p<0.001).

**Figure 3:**
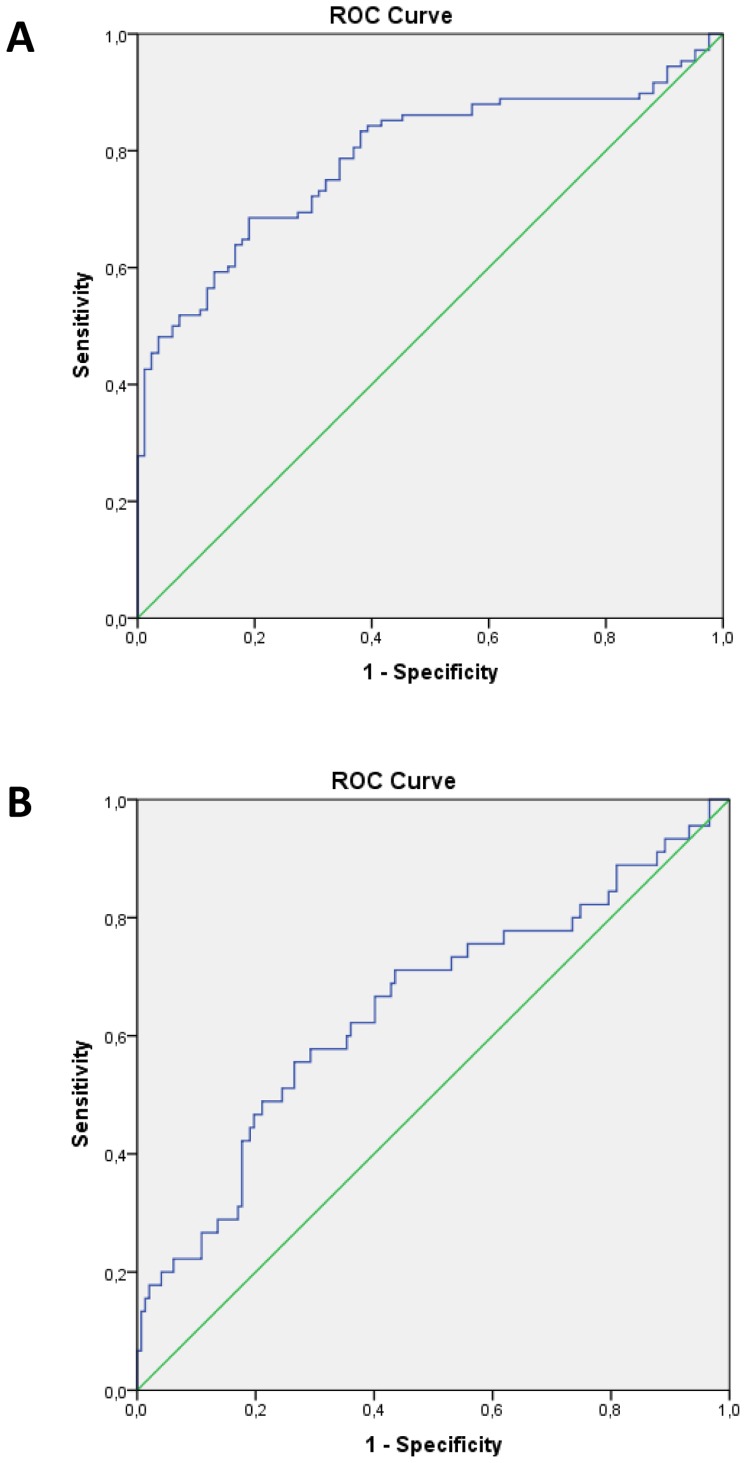
ROC-curves for analysis of sensitivity/specificity ratio to evaluate diagnostic significance of integral mutation burden of mtDNA, when the presence of any atherosclerotic plaque in any visualized carotid segment (A), or the presence of CHD (B) are taken as actual states. Integral mutation burden is calculated by using logistic regression model, which predicted the probability of belonging to a particular category (the predictors are the levels of heteroplasmy for C3256T, T3336C, G12315A, G13513A, G14459A, G14846A, and G15059A mutations).

The ROC-curve analysis of sensitivity/specificity ratio was performed to evaluate diagnostic significance of mutation burden of mtDNA, when the presence of any atherosclerotic plaque in any visualized carotid segment (Fig. 3-A), or the presence of CHD (Fig. 3-B) were taken as actual states. Integral mutation burden was calculated by using logistic regression model, which predicted the probability of belonging to a particular category (the predictors were the levels of heteroplasmy for C3256T, T3336C, G12315A, G13513A, G14459A, G14846A, and G15059A mutations). For the presence of atherosclerotic plaque, the area under curve accounted for 0.788±0.033, p<0.001 (95% confidence interval, 0.724–0.853). The cut-off value for integral mutation burden accounted for 0.5558; under these conditions, the sensitivity of this measure was 72.2%, and specificity was 70.2%. For the presence of CHD, the area under curve accounted for 0.648±0.050, p = 0.003 (95% confidence interval, 0.549–0.746). The cut-off value for integral mutation burden accounted for 0.5558; under these conditions, the sensitivity of this measure was 62.2%, and specificity was 63.9%.

The other kind of ROC-curve analysis, when integral mutation burden calculated on the basis of quartile numbers of heteroplasmies distribution was taken as test variable, and the presence of any atherosclerotic plaque in any visualized segment of carotid arteries was taken as an actually observed state, has shown that the area under curve accounted for 0.707±0.038 (95% confidence interval, 0.632–0.781). The cut-off value for integral mutation burden accounted for 1.5; under these conditions, the sensitivity of this measure was 63.0%, and specificity was 63.1%.

The relationship between the levels of heteroplasmy of mtDNA and the presence of CHD in study participants was also examined. The results are presented in Table 2. The levels of heteroplasmy for C3256T, T3336C, and G12315A were significantly higher, and for G13513A and G14459A were significantly lower in CHD patients as compared to study participants without clinical manifestations of atherosclerosis. Seven CHD patients also had the history of an acute myocardial infarction; the levels of heteroplasmy for C3256T and G12315A mutations in them were significantly higher as compared to the other study participants (38.4%, SD 20.7, vs 22.7%, SD 14.2, p = 0.041, and 48.0%, SD 18.5, vs 32.1%, SD 19.3, p = 0.043).

In spite of relatively small sample size, statistical power in this study reached 100% for the presence of carotid atherosclerotic plaques in any visualized segment of carotid arteries, 89% for the presence of CHD, and only 60% for the history of myocardial infarction (all at α<0.05). This trend is easily explained by the fact that clinical manifestations like CHD and myocardial infarction should be considered as a probability function of the presence of atherosclerosis itself, which is an obligatory prerequisite for clinically significant consequences. It may be speculated that in the given time individuals already have subclinical atherosclerosis, which is in part explained by mutations of mtDNA; to evaluate prognostic significance of proatherogenic and atheroprotective mtDNA mutations, long-tern prospective studies are needed. Our data demonstrate that the presence of atherosclerosis and CHD is associated significantly with integral mutation burden of mitochondrial genome. Therefore, the assessment of impact of single mutation should be made on the basis of weighed coefficients obtained from regression analysis. The thresholds for the level of heteroplasmy for mutation to be in contention as a marker were obtained from ROC-analysis. For G12315A it accounted for 26.5%; this value provided the sensitivity 0.704, and specificity 0,607; however, a range of values can be considered.

## Discussion

Earlier we demonstrated a relation between C3256T and predisposition to atherosclerosis [20]. This mutation is located in coding sequence of the MT-TL1 gene (codon recognizing UUR) which encodes tRNA leucine [21]–[23], and is expressed at the cellular level as a reduced amount of cellular organelles and impaired protein synthesis [21]–[23]. In this study we show for the first time that at least six of other mitochondrial mutations (T3336C, G12315A, G13513A, G14459A, G14846A, and G15059A) are also associated with the extent of carotid atherosclerosis, which was diagnosed in our study by means of quantitative ultrasound examination of carotid arteries.

One of newly studied mutation, namely G12315A mutation which can be expressed at the cellular level as a altered amount of cellular organelles and impaired protein is located in the coding sequence of mitochondrial gene encoding tRNA leucine, the MT-TL2 gene (codon recognizing CUN). The results of this study demonstrate that this mutation is associated with the extent of atherosclerosis and even with its clinical manifestation CHD and, possibly, myocardial infarction. Thus, the impairments of tRNA leucine synthesis may act as a previously unknown mechanism for atherosclerosis development and progression. By now, G12315A mutation is described to be associated with mitochondrial encephalomyopathy [8], [24], [25]. The association of this mutation with atherosclerosis has not been reported previously. Another studied mutation T3336C is located in the coding region of the MT-ND1 gene encoding subunit 1 of NADH dehydrogenase; however, it is considered to be a silent point mutation producing no changes in protein sequence (ATT → ATC, Ile). We suggest, however, that this mutation may be associated e.g. in linkage with some mutant haplotype, still unknown, which produces human pathologies. This assumption is partially supported by the high correlation of heteroplasmy levels of T3336C and G12315A mutations, the latter of which was strongly atherogenic in the present data.

Mutations G13513A, G14459A, G14846A, and G15059A occur in coding regions of genes responsible for the synthesis of respiratory chain enzymes (MT-ND5 and MT-ND6 genes encoding the subunits 5 and 6 of NADH dehydrogenase, respectively, and MT-CYB gene encoding cytochrome B). An impairment of NADH dehydrogenase activity can be expected to attenuate NADH oxidation and CoQ (ubiquinone) reduction and thus promote oxidative stress. Mutation G13513A (MT-ND5 gene) is believed to be associated with hereditary encephalomyopathy, cardiomyopathy, and the WPW syndrome [25]–[28]. Mutation G14459A (MT-ND6 gene) results in alanine to valine substitution in a conserved region of ND6 protein at position 72, and is associated with hereditary ocular neuropathy, atrophy of visual nerve, Leber’s hereditary visual neuropathy, dysfunction of basal ganglia, musculospastic syndrome and encephalopathy [13], [29]–[31]. Mutations G14846A and G15059A may lead to the damage of cytochrome B: the first one results in glycine to serine substitution in position 34, thus affecting intermediate transfer of electrons in mitochondrial respiratory chains; the second one results in glycine to stop codon substitution at position 190, thus stopping translation and leading to the loss of 244 amino acids at C-terminal of protein. Both mutations are capable of reducing enzymatic function of cytochrome B, and associated with mitochondrial myopathies [1], [32], [33].

The fact that none of the mutations examined in our study have been yet associated with either atherosclerosis or CHD might be due, in part, to methodological issues: the vast majority of the existing nucleotide sequence analysis methods do not allow precise quantitative measurement of heteroplasmies but indicate only the presence of the mutant allele or provide semi-quantitative assessment of proportion of mutant alleles in biological samples. We demonstrated in the present study a high prevalence of mutations of mitochondrial genome in a population sample, in which the participants had no clinical signs of any mitochondrial disease. Moreover, it is known nowadays that pathogenic mitochondrial DNA mutations are very common in the general population [34], [35].Obviously, clinical and phenotypic manifestations should depend on the levels of heteroplasmies.

Surprisingly, high prevalence of mitochondrial mutations, as well as rather high levels of heteroplasmies was found in this study; it may be supposed that such phenomenon should have been detected in the many hundreds of mtDNA genomes sequenced in the past by Sanger sequencing for other purposes. However, direct sequencing is not the method of choice for the quantitation of heteroplasmy levels. As a rule, sequence analysis of heteroplasmy around 50% provides clear results in terms of the presence of heteroplasmy, but not in a quantitative manner. Lower level heteroplasmy is often undetectable by direct sequencing. As an example, Meierhofer et al. have used denaturing high performance liquid chromatography to rapidly screen the entire mtDNA for mutations; this approach yielded straightforward interpretation of results with a detection limit down to 1% mtDNA heteroplasmy. However, direct sequencing analysis has become informative only after collection and re-amplification of low degree heteroduplex peak-fractions [36]. Moreover, recently we have performed full mtDNA sequencing using NGS approach (Roche’s 454 Sequencing technique) in 30 randomly selected persons. We have detected 160 novel mutations of mtDNA, which have not been described previously in numerous studies on direct mtDNA sequencing; among them, 24 mutations were detected in 10–60% cases, in which the heteroplasmy level varied from 7% to 64% (preliminary data, not published). This finding provides one more evidence that mitochondrial DNA mutations are much more common in population, than could be expected from earlier knowledge.

Mitochondrial mutations can be either somatic or inherited through the maternal line. They are characterized by the phenomenon of heteroplasmy, which is defined as the presence of a mixture of more than one type of an organellar genome within a cell or individual. Mitochondrial DNA is present in hundreds to thousands of copies per cell and also has a very high mutation rate. New mtDNA mutations arise in cells, coexist with wild-type mtDNA, and segregate randomly during cell division. The high prevalence of the examined mutations of mitochondrial genome suggests that they are maternally inherited. On the other hand, the heteroplasmy levels of six out of 10 mutations rose with increasing age, which supports also the somatic nature of mutations: in any case, there is an increase in the proportion of mutant alleles of the mitochondrial genome of human white blood cells with age. Although it appears likely, it is not known whether the processes of accumulation of the mutant allele occur in other tissues of the human body. Preferential survival of somatic cells or progenitor cells with higher content of the mutant allele in the mitochondrial DNA cannot be excluded either, although this assumption contradicts the association of mitochondrial mutations with neurodegenerative diseases and atherosclerosis, which reduce longevity.

Leukocytes play a special role in atherogenesis [37]. They migration of variety of leukocyte subtypes in the subendothelial layer in arteries and their participation in the processes of inflammation and atherosclerotic plaque formation is well documented [38]. It is possible to expect that a high level of mtDNA heteroplasmy in cells that circulate in the blood stream might be indicative of a high likelihood that the defective leukocytes with impaired mitochondrial function would enter into the arterial intimal layer. If leukocyte function is inhibited due to the presence of mutations in coding regions of mtDNA, this may lead to local oxidative stress and other pathologic events which could promote atherosclerosis formation. Thus one can assume that mtDNA heteroplasmy, being a biomarker of defective mitochondrial function in leukocytes, can also be regarded as a biomarker for atherosclerosis and consequent clinical manifestations such as CHD.

The findings of the present investigation open prospects for further studies. Even though the sample size in the present investigation was sufficient to detect significant differences in the levels of heteroplasmy of mitochondrial genome between non-atherosclerotic participants and patients with subclinical atherosclerosis, or between CHD-free individuals and CHD patients, the results of this study may be needed to be extended by the use of population sample taken not only from ethnically heterogeneous population of Moscow that consisted of senior and elderly ages but also from inhabitants of younger age. Obviously, other ethnic groups should be studied as well. Finally, it is worth to noting here that the present study was cross-sectional and thus the assessment of actual risk of atherosclerosis and cardiovascular disease due to the presence of mutations of mitochondrial genome requires further prospective studies.

The findings of the study indicate that heteroplasmies for several mutations in the mtDNA in leukocytes, including C3256T, T3336C, G12315A, G13513A, G14459A, G14846A, and G15059A mutations, are significantly (p<0.001) associated with both the severity of carotid atherosclerosis and the presence of CHD. These findings suggest that somatic mitochondrial mutations might have a role in the development of atherosclerosis.

## Materials and Methods

This study was kept in accordance with the Helsinki Declaration of 1975 as revised in 1983. It was approved by the local ethics committees of the Institute of General Pathology and Pathophysiology, Moscow, and Institute for Atherosclerosis Research, Skolkovo Innovation Center, Moscow, Russia. All participants gave their written informed consent prior to their inclusion in the study.

The study participants were recruited consecutively from the visitors flow at Moscow municipal outpatient clinics No. 202, who have passed a routine screening for cardiovascular risk factors (mainly blood cholesterol and arterial blood pressure). Exclusion criteria were anatomic configuration of neck and carotid arteries preventing from qualitative ultrasonography, serious life-threatening diseases, and the refusal from signing informed consent form. In total, 190 participants were recruited (84 men, 106 women) aged 65.0 years (SD 9.4); among them 45 participants (24%) had clinical CHD. The gender ratio was similar in healthy participants and CHD patients (P = 0.17). CHD patient were older than healthy participants; mean age was 70.0 (SD 8.7) and 63.5 (SD 9.0), respectively, P<0.001. Antropometric, clinical and biochemical characteristics of study participants are given in Table 4.

**Table 4:** Antropometric, clinical and biochemical characteristics of study participants.

Variable	Total group, n = 190	Non-CHD controls, n = 145	CHD patients, n = 45	P for the difference
Age, years	65.0 (9.4)	63.5 (9.0)	70.0 (8.7)	<0.001 *
Gender, m:f	84∶106	60∶85	24∶21	NS (0.10)
BMI, kg/m^2^	26.9 (4.6)	26.6 (4.5)	27.7 (4.8)	NS (0.10) **
SBP, mm Hg	139 (17)	138 (17)	141 (18)	NS *
DBP, mm Hg	83 (11)	83 (10)	81 (13)	NS ***
Smokers, %	8.9	11.0	2.2	NS (0.06)
Hypertension, %	64.9	58.9	84.4	0.001
LVH, %	34.0	28.1	53.3	0.002
Diabetes, %	12.6	6.8	31.1	<0.001
Angina, %	24.1	0.0	100.0	<0.001
AMI in anamnesis, %	3.7	0.0	15.6	<0.001
Stroke in anamnesis, %	2.6	0.0	8.9	0.011
Family anamnesis for AMI, %	27.7	26.0	33.3	NS
Family anamnesis for HT, %	39.3	39.0	40.0	NS
Family anamnesis for T2DM, %	17.3	17.1	17.8	NS
Total cholesterol, mg/dl	239 (48)	240 (48)	235 (50)	NS *
Triglycerides, mg/dl	127 (60)	125 (60)	131 (60)	NS **
LDL cholesterol, mg/dl	148 (43)	148 (43)	145 (45)	NS *
HDL cholesterol, mg/dl	66 (15)	67 (15)	63 (16)	NS *
Fasting blood glucose, mmol/l	4.9 (1.2)	4.8 (1.2)	5.2 (1.6)	NS *
Statins, %	11.5	7.5	24.4	0.006
Plaque, score	0.83 (0.86)	0.73 (0.85)	1.16 (0.85)	0.003 **
Mean cIMT, µm	869 (167)	841 (150)	961 (189)	<0.001 *
Maximum cIMT, µm	1006 (213)	971 (185)	1117 (258)	<0.001 **

* one-way ANOVA;

** Mann-Whitney U-test;

*** Welch test.

High-resolution B-mode ultrasonography was used for carotid arterial imaging to assess the extent of carotid atherosclerosis. The protocol of ultrasound examination involved the scanning of the right and left common carotid artery and the area of the carotid sinus (bulb) as high up as possible [39]. Three fixed angles of interrogation were used (anterolateral, lateral, and posterolateral). Images were focused on the posterior wall of the artery. The B-mode ultrasound system (SSI-1000, SonoScape, China) used a 7.5 MHz linear array probe. The measurements were always performed at 10-mm section of common carotid artery adjacent to the carotid bulb. The carotid intima-media thickness (cIMT) of the posterior wall was measured as the distance from the leading edge of the first echogenic (bright) line to the leading edge of the second echogenic line. The measurements were carried out with M’Ath computer software (IMT, France). The mean of three measurements (in anterolateral, lateral, and posterolateral positions) was considered to be the integral cIMT estimate. Reproducibility of cIMT measurements was assessed according to the protocol of the IMPROVE Study [40].

The degree of susceptibility to atherosclerosis was estimated by using interquartile cIMT values derived from Moscow population sample of 1287 participants (429 men, 858 women) free of CHD (Table 5). Such approach allowed distinguishing persons predisposed or not predisposed to atherosclerosis. If a person belonged to the lowest quartile of age-adjusted cIMT distribution, this person was considered as non-predisposed to atherosclerosis; if the person belonged to the highest quartile, then this person had a high predisposition to atherosclerosis. The belonging to the 2nd or the 3rd quartiles of cIMT distribution was regarded as a moderate or elevated susceptibility, respectively.

**Table 5:** Interquartile cIMT values derived from Moscow population.

	Age			
	<50	51–60	61–70	>70
**Men**				
2nd quartile, µm	660	740	830	840
3rd quartile, µm	745	810	910	930
4th quartile, µm	800	910	990	1060
**Women**				
2nd quartile, µm	605	665	760	825
3rd quartile, µm	665	735	830	895
4th quartile, µm	740	815	920	990

Additionally, the presence and the size of atherosclerotic plaques in any visualized segment of carotid arteries was evaluated by a 4-point scale (0 - no atherosclerotic lesions; 1–2 - elevated atherosclerotic plaques taking up to 20% or 20 to 50% of lumen diameter, respectively: 3 - hemodynamically significant atherosclerotic plaques taking more than 50% of lumen diameter).

DNA samples were obtained from whole venous blood using a commercially available kit for DNA purification (QIAGEN GmbH, Germany). For the amplification of fragments of mitochondrial DNA by polymerase chain reaction (PCR) method followed by pyrosequencing, the previously described primers and conditions were used [18]. In brief, to quantitatively evaluate mutant allele, a method of pyrosequencing [41]–[43] was adapted for conditions where both normal and mutant alleles were present in a biological specimen; the defective allele was quantified by analyzing the peak heights in the pyrogram of one-chained PCR-fragments of a mitochondrial genome. The levels of heteroplasmy in DNA samples were calculated, taking into account the expected sequence and the dimension of peaks for the homozygotes possessing either 100% of the normal or 100% of the mutant allele, as described elsewhere [18]. The nucleotide sequences for forward primers, reverse primers, and sequence primers are represented in Table 6.

**Table 6:** Primers for PCR and pyrosequencing.

Mutation	Forward primer for PCR	Reverse primer for PCR	Sequence primer
G1555A	TAGGTCAAGGTGTAGCCCATGAGGTGGCAA	bio-GTAAGGTGGAGTGGGTTTGGG	ACGCATTTATATAGAGGA
C3256T	bio-AGGACAAGAGAAATAAGGCC	ACGTTGGGGCCTTTGCGTAG	AAGAAGAGGAATTGA
T3336C	bio-AGGACAAGAGAAATAAGGCC	ACGTTGGGGCCTTTGCGTAG	TGCGATTAGAATGGGTAC
C5178A	bio-GCAGTTGAGGTGGATTAAAC	GGAGTAGATTAGGCGTAGGTAG	ATTAAGGGTGTTAGTCATGT
G12315A	bio-CTCATGCCCCCATGTCTAA	TTACTTTTATTTGGAGTTGCAC	TTTGGAGTTGCAC
G13513A	CCTCACAGGTTTCTACTCCAAA	bio-AAGTCCTAGGAAAGTGACAGCGAGG	AGGTTTCTACTCCAA
G14459A	CAGCTTCCTACACTATTAAAGT	bio-GTTTTTTTAATTTATTTAGGGGG	GATACTCCTCAATAGCCA
G14846A	CAGCTTCCTACACTATTAAAGT	bio-GTTTTTTTAATTTATTTAGGGGG	GCGCCAAGGAGTGA
G15059A	CAGCTTCCTACACTATTAAAGT	bio-GTTTTTTTAATTTATTTAGGGGG	TTTCTGAGTAGAGAAATGAT

Within this study, a total of 43 mutations of mtDNA have been tested (A1555G, A3280G, A750G, C14482C, C14482G, C15452A, C3256T, C3285T, C5178A, C6489A, G12351A, G13513A, G14459A, G14846A, G15059A, G15084A, G15762A, G3316A, G5540A, G8363A, G3316A, G9379A, T14484C, T14487C, T14709C, T3258C, T3271C, T3336C, T5692C, T5814C, T716G, T8362G, T8993C, T8993G, insertions ins5132AA, ins652G, ins961C, and deletions del15498_23, del5132AA, del652G, del9489, del9537C, del961C). As a result, most of mutations were excluded from further analysis, as they were not found in DNA from leukocytes, or have demonstrated a negligibly low level of heteroplasmy (1–5%). Mutations C3256T, T3336C, G1231A, G13513A, G14459A, G14846A and G15059A were found to have high prevalence in the study sample, and demonstrated high variability; therefore, they were selected for further analysis. The results on C3256T heteroplasmy seemed to have the highest diagnostic significance; therefore, we have analyzed and reported the data on C3256T separately [20]. However, during in-depth analysis several mutations proved to be associated in some way either with cIMT, or CHD, or the presence of atherosclerotic plaques. Thus, the role of integral mutation burden of mtDNA in predisposition to atherosclerosis or its clinical manifestations has been analyzed, and within this analysis, C3256T heteroplasmy was included as one of the factors responsible for formation of integral mutation burden.

As the validity of the heteroplasmy measurements seemed to be crucial, and pyrosequencing method is not so common and self-explanatory for the reader, an experimental proof with the introduction of different proportion of a mutated allele was obtained. Theoretical background for calculation of heteroplasmy level by analyzing the peak heights in the pyrogram, as well as original peak height histograms of real samples were described elsewhere [18]. To get the data on pyrosequencing calibration, the mixtures of DNA samples with either 100% of the normal or 100% of the mutant allele were tested, with the ratio of 1∶0 (homoplasmy), 4∶1 (20% heteroplasmy), 2∶3 (40% heteroplasmy), 1∶2 (67% heteroplasmy), and 0∶1 (homopasmy). To estimate the reproducibility of measurements, this set of DNA mixtures was analyzed in 6 independent experiments. The original pyrograms are shown at Figure S1. The results of measurements were very close to the expected values, and accounted for 0%, 18%, 42%, 66%, and 100%. The data on reproducibility of measurements are given in Table S1. The mean value of coefficient of variation for 20%, 40% and 67% heteroplasmic mixtures accounted for 4.7%, and for 100% mutant allele –2.1%; in the opposite, for 100% normal allele small erroneous peaks for mutant nucleotide were detected, which provided a coefficient of variation of 35.1% (Figure S2). These observations allowed us to conclude that for determining the level of heteroplasmy higher than 10%, the coefficient of variation does not exceed 10%, and the results of quantitative heteroplasmy measurements are accurate and reproducible. However, for very low heteroplasmy levels (less than 5%), this method is effective for the assessment of the presence of heteroplasmy, but does not provide precise measurements. On the other hand, such low heteroplasmy levels will hardly result in clinical consequences. Based on this assumption, the heteroplasmy levels below 5% were considered negligible.

To demonstrate the variability in heteroplasmy levels in real DNA samples, several original pyrograms are presented in Figure S3.

Statistical analysis was performed using the SPSS 14.0 software (SPSS Inc., USA). The methods of one-way analysis of variance, cross-tabulation analysis, and correlation analysis by Spearman and Pearson were used. The comparison of mean values for continuous variables was performed using the U-test by Mann-Whitney, for categorical variables by chi-square Pearson’s test. The data are presented in terms of mean and SD. The significance of differences was defined at the 0.05 level of confidence.

## Supporting Information

Figure S1 Pyrograms of the mixtures of DNA samples with the ratio of normal and mutant allele 1∶0 (homoplasmy, 0% mutant allele), 4∶1 (20% heteroplasmy), 2∶3 (40% heteroplasmy), 1∶2 (67% heteroplasmy), and 0∶1 (homopasmy, 100% mutant allele).
(TIF)

Figure S2 Graph showing the relationship between the level of heteroplasmy in mixed DNA samples and coefficient of variation of measurements.
(TIF)

Figure S3 Practical pyrograms for the measurement of mtDNA heteroplasmy levels. Upper row, C3256T heteroplasmy; left –4% heteroplasmy, right –13% heteroplasmy. Middle row, G12315A heteroplasmy; left –0% heteroplasmy, right –83% heteroplasmy. Lower row, G13513A heteroplasmy; left –4% heteroplasmy, right –74% heteroplasmy.
(TIF)

Table S1 The descriptive statistics and analysis of reproducibility of heteroplasmy level measurements by pyrosequensing method in DNA mixtures.
(DOCX)

Text File S1 **TEM procedures.** For electron microscopic analysis, samples of white blood cells were processed according to the procedures described by James *et al* [1]. As a fixative, 1.5% glutaraldehyde in 0.1 M phosphate buffer (pH 7.2) was used; for post-fixation 1% OsO_4_ was used. White blood cells were embedded in Araldite resin. Ultrathin sections were stained with uranyl acetate and lead citrate and examined with the aid of a Hitachi H7000 electron microscope at an accelerating voltage of 75 kV. 1. James V, Winfield DA, James N (1988) Ultrastructural features of acute monoblastic leukaemia cells: a multivariate morphometric analysis. Virchows Arch A Pathol Anat Histopathol 414∶21–27.
(DOCX)
