# Supplementary figures and images for: Association of Mitochondrial Genetic Variation with Carotid Atherosclerosis
Source: PLoS One. 2013 Jul 9;8(7):e68070. doi: 10.1371/journal.pone.0068070 (PMC3706616; doi:10.1371/journal.pone.0068070)

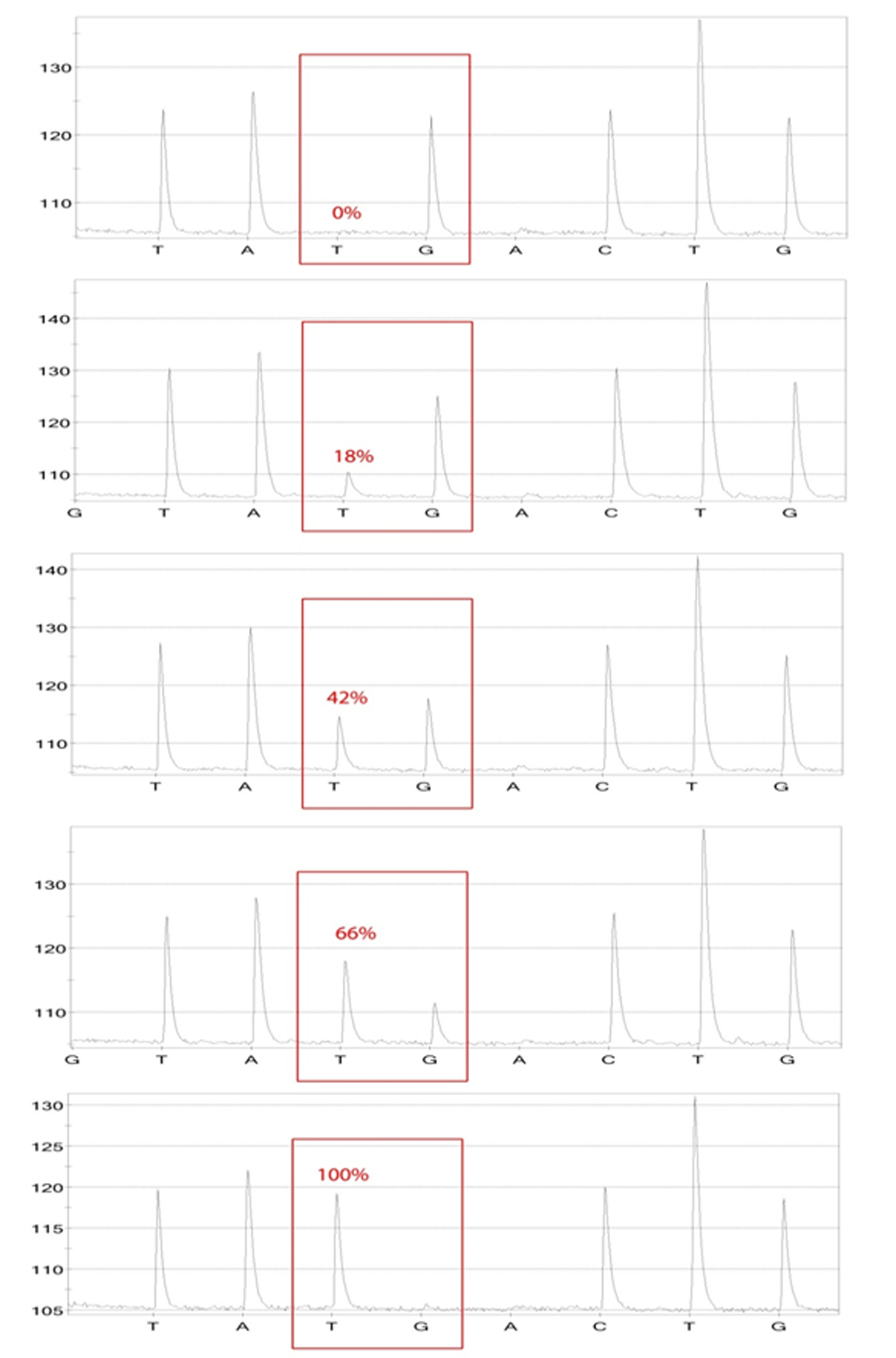

Supplement: Figure S1 — Pyrograms of the mixtures of DNA samples with the ratio of normal and mutant allele 1∶0 (homoplasmy, 0% mutant allele), 4∶1 (20% heteroplasmy), 2∶3 (40% heteroplasmy), 1∶2 (67% heteroplasmy), and 0∶1 (homopasmy, 100% mutant allele). (TIF) [file pone.0068070.s001.tif]

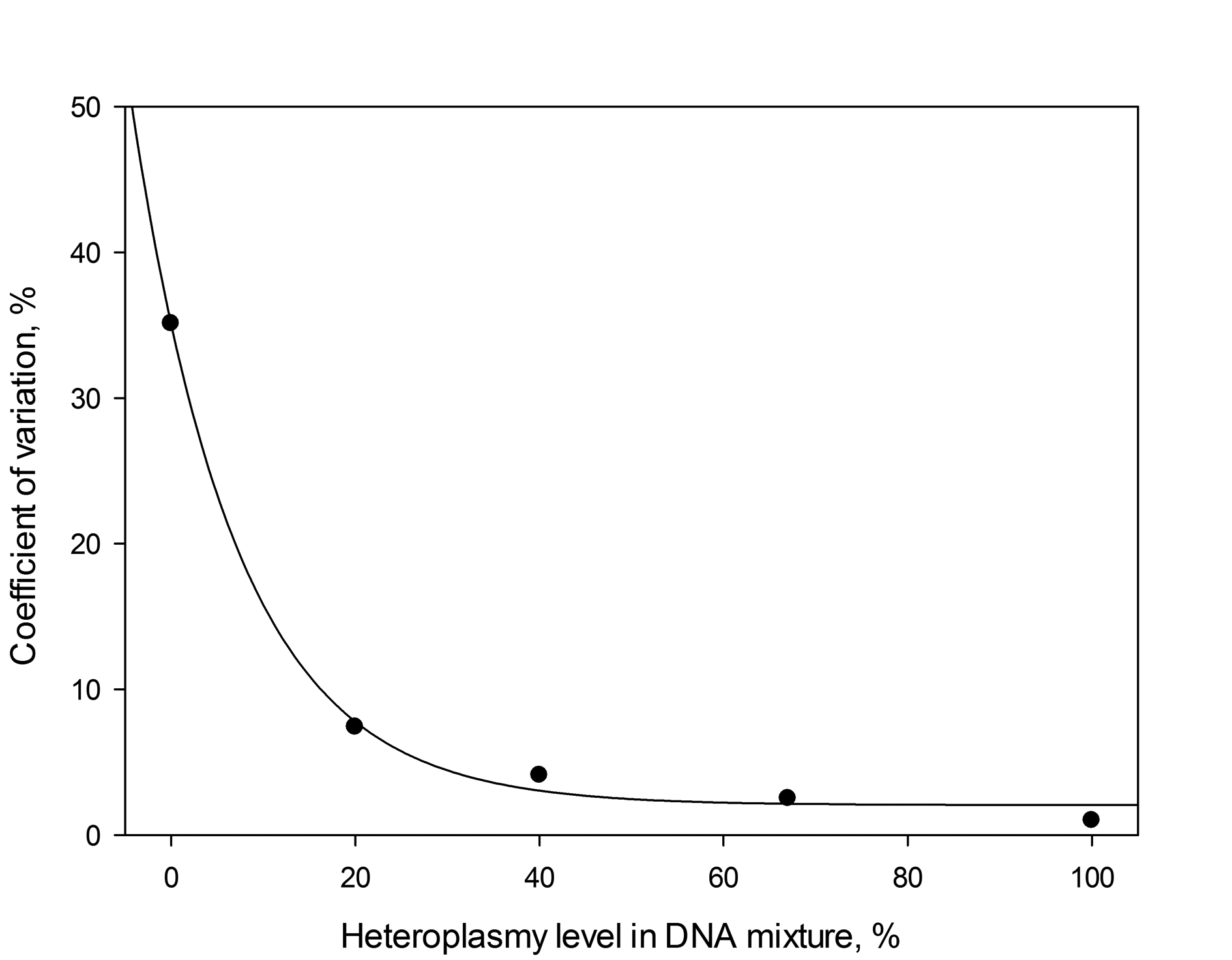

Supplement: Figure S2 — Graph showing the relationship between the level of heteroplasmy in mixed DNA samples and coefficient of variation of measurements. (TIF) [file pone.0068070.s002.tif]

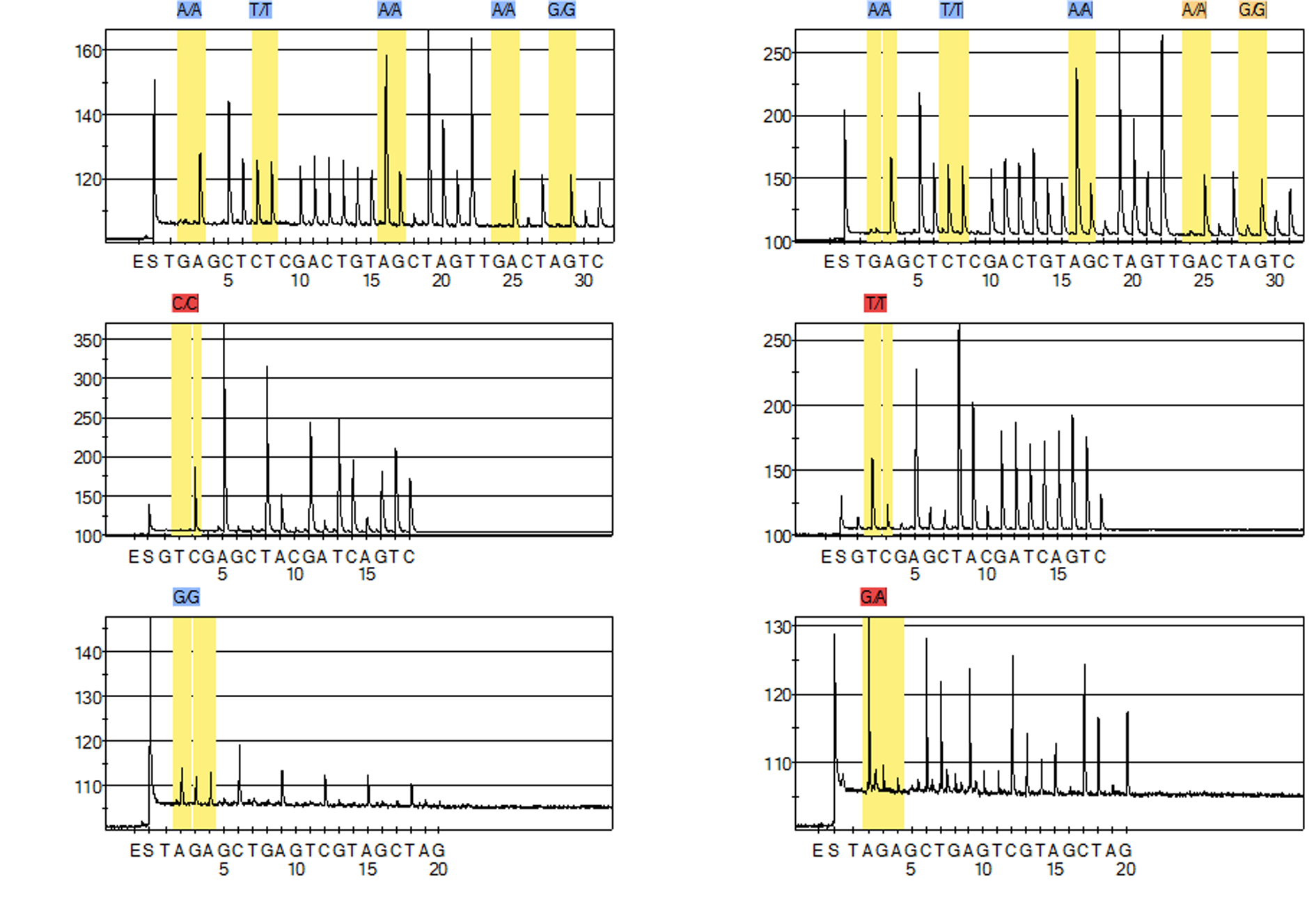

Supplement: Figure S3 — Practical pyrograms for the measurement of mtDNA heteroplasmy levels. Upper row, C3256T heteroplasmy; left –4% heteroplasmy, right –13% heteroplasmy. Middle row, G12315A heteroplasmy; left –0% heteroplasmy, right –83% heteroplasmy. Lower row, G13513A heteroplasmy; left –4% heteroplasmy, right –74% heteroplasmy. (TIF) [file pone.0068070.s003.tif]
