# Supplementary material for: Association of Mitochondrial Genetic Variation with Carotid Atherosclerosis
Source: PLoS One. 2013 Jul 9;8(7):e68070. doi: 10.1371/journal.pone.0068070 (PMC3706616; doi:10.1371/journal.pone.0068070)
Supplement: Table S1 — The descriptive statistics and analysis of reproducibility of heteroplasmy level measurements by pyrosequensing method in DNA mixtures. (DOCX) [file pone.0068070.s004.docx]

**Table S1**. The descriptive statistics and analysis of reproducibility of heteroplasmy level measurements by pyrosequensing method in DNA mixtures.

| **DNA mixture sample** | **Number of measurements, n** | **Heteroplasmy level, %** | | | **SD** | **Δ mean** | **CV, %** |
| --- | --- | --- | --- | --- | --- | --- | --- |
|  |  | Minimum | Maximum | Mean |  |  |  |
| 1:0 (100% normal allele) | 6 | 0.3 | 1.1 | 0.77 | 0.27 | 0.27 | 35.1 |
| 1:4 (20% heteroplasmy) | 6 | 15.5 | 19.5 | 17.91 | 1.56 | 1.33 | 7.4 |
| 2:3 (40% heteroplasmy) | 6 | 40.0 | 44.0 | 42.11 | 1.44 | 1.72 | 4.1 |
| 1:2 (67% heteroplasmy) | 6 | 64.1 | 67.1 | 65.61 | 1.02 | 1.61 | 2.5 |
| 0:1 (100% mutant allele) | 6 | 97.5 | 98.9 | 98.27 | 0.57 | 0.97 | 1.0 |
