# Supplementary material for: Association of Mitochondrial Genetic Variation with Carotid Atherosclerosis
Source: PLoS One. 2013 Jul 9;8(7):e68070. doi: 10.1371/journal.pone.0068070 (PMC3706616; doi:10.1371/journal.pone.0068070)
Supplement: Text File S1 — TEM procedures. For electron microscopic analysis, samples of white blood cells were processed according to the procedures described by James et al [1]. As a fixative, 1.5% glutaraldehyde in 0.1 M phosphate buffer (pH 7.2) was used; for post-fixation 1% OsO4 was used. White blood cells were embedded in Araldite resin. Ultrathin sections were stained with uranyl acetate and lead citrate and examined with the aid of a Hitachi H7000 electron microscope at an accelerating voltage of 75 kV. 1. James V, Winfield DA, James N (1988) Ultrastructural features of acute monoblastic leukaemia cells: a multivariate morphometric analysis. Virchows Arch A Pathol Anat Histopathol 414∶21–27. (DOCX) [file pone.0068070.s005.docx]

**Text file S1**

*TEM procedures*

For electron microscopic analysis, samples of white blood cells were processed according to the procedures described by James *et al* [1]. As a fixative, 1.5% glutaraldehyde in 0.1 M phosphate buffer (pH 7.2) was used; for post-fixation 1% OsO_4_ was used. White blood cells were embedded in Araldite resin. Ultrathin sections were stained with uranyl acetate and lead citrate and examined with the aid of a Hitachi H7000 electron microscope at an accelerating voltage of 75 kV.

1. James V, Winfield DA, James N (1988) Ultrastructural features of acute monoblastic leukaemia cells: a multivariate morphometric analysis. Virchows Arch A Pathol Anat Histopathol 414: 21-27.
